# Adipose-Derived Stromal Cell-Sheets Sandwiched, Book-Shaped Acellular Dermal Matrix Capable of Sustained Release of Basic Fibroblast Growth Factor Promote Diabetic Wound Healing

**DOI:** 10.3389/fcell.2021.646967

**Published:** 2021-03-25

**Authors:** Xin Shi, Liyuan Jiang, Xin Zhao, Bei Chen, Wei Shi, Yanpeng Cao, Yaowu Chen, Xiying Li, Yusheng He, Chengjie Li, Xiaoren Liu, Xing Li, Hongbin Lu, Can Chen, Jun Liu

**Affiliations:** ^1^The First School of Clinical Medicine, Southern Medical University, Guangzhou, China; ^2^Department of Limbs (Foot and Hand) Microsurgery, Affiliated Chenzhou Hospital, Southern Medical University, Chenzhou, China; ^3^Key Laboratory of Organ Injury, Aging and Regenerative Medicine of Hunan Province, Changsha, China; ^4^Hunan Engineering Research Center of Sports and Health, Changsha, China; ^5^Department of Spine Surgery, Xiangya Hospital, Central South University, Changsha, China; ^6^Department of Emergency, Hubei Provincial Hospital of Traditional Chinese Medicine, Wuhan, China; ^7^Department of Orthopedics, Xiangya Hospital, Central South University, Changsha, China; ^8^Department of Sports Medicine, Xiangya Hospital, Central South University, Changsha, China; ^9^The First School of Clinical Medicine, Xiangnan University, Chenzhou, China

**Keywords:** adipose-derived stromal cells, decellularized dermal matrix, fibroblast growth factor, diabetic wound healing, collagen-binding domain

## Abstract

The management of diabetic wounds is a therapeutic challenge in clinical settings. Current tissue engineering strategies for diabetic wound healing are insufficient, owing to the lack of an appropriate scaffold that can load a large number of stem cells and induce the interaction of stem cells to form granulation tissue. Herein we fabricated a book-shaped decellularized dermal matrix (BDDM), which shows a high resemblance to native dermal tissue in terms of its histology, microstructure, and ingredients, is non-cytotoxic and low-immunogenic, and allows adipose-derived stromal cell (ASC) attachment and proliferation. Then, a collagen-binding domain (CBD) capable of binding collagen was fused into basic fibroblast growth factor (bFGF) to synthetize a recombinant growth factor (termed as CBD–bFGF). After that, CBD–bFGF was tethered onto the collagen fibers of BDDM to improve its endothelial inducibility. Finally, a functional scaffold (CBD–bFGF/BDDM) was fabricated. *In vitro* and *in vivo* experiments demonstrated that CBD–bFGF/BDDM can release tethered bFGF with a sustained release profile, steadily inducing the interaction of stem cells down to endothelial differentiation. ASCs were cultured to form a cell sheet and then sandwiched by CBD–bFGF/BDDM, thus enlarging the number of stem cells loaded into the scaffold. Using a rat model, the ASC sheets sandwiched with CBD–bFGF/BDDM (ASCs/CBD–bFGF/BDDM) were capable of enhancing the formation of granulation tissue, promoting angiogenesis, and facilitating collagen deposition and remodeling. Therefore, the findings of this study demonstrate that ASCs/CBD–bFGF/BDDM could be applicable for diabetic wound healing.

## Introduction

As a worldwide health concern, diabetes affects approximately 420 million people (American Diabetes and Association, 2013; Cho et al., 2019; Li et al., 2020). Diabetic foot ulcers (DFUs), a major complication of diabetes mellitus and a type of chronic wound, occur in 15–25% of patients with diabetes (Shrestha et al., 2013; Liu et al., 2017; Atosona and Larbie, 2019; Grennan, 2019). More seriously, greater than 14–24% of these patients will require amputation as a result of progressive disease (Okonkwo and DiPietro, 2017; Grennan, 2019). The current standard of treatment includes debridement of the wound, infection control, and application of various wound dressings to facilitate healing (Brem et al., 2004; Wu et al., 2007). Regrettably, in many patients, these curative treatments are not efficient in facilitating rapid wound healing; thus, non-healing diabetic ulcers cause an extremely heavy burden on their families and the healthcare system (2013; Zhang et al., 2020). Therefore, it is urgent to develop an affordable and efficacious treatment strategy.

Over the past decades, the emergence and development of tissue engineering has provided a promising treatment strategy for diabetic wound healing, utilizing a combination of scaffolds, seeding cells, and growth factors (GFs) to replace or repair wounded skin (Rahmani Del Bakhshayesh et al., 2018; Cho et al., 2019; Nour et al., 2020; Yang et al., 2020). Among these three factors, scaffolds can be fundamentally essential since they provide structural support and signals to modulate cellular responses for tissue regeneration (Cho et al., 2019). In recent years, decellularized scaffolds have attracted wide attention in tissue engineering, owing to their low immunogenicity, high biocompatibility, good biodegradability, and high similarity to target tissue in terms of their morphology and ingredients (Cheng et al., 2014; Jakus et al., 2017; Chen et al., 2019). More importantly, decellularized scaffolds can provide a natural three-dimensional environment with tissue-specific extracellular matrix (ECM) that can directly regulate the migration, proliferation, and even differentiation of the attached cells (Cheng et al., 2014; Jakus et al., 2017; Chen et al., 2019). Currently, clinical studies have shown that several kinds of commercial decellularized dermal matrix (DDM) can significantly decrease the wound area and promote the healing of DFUs (Guo et al., 2017; Zelen et al., 2018). However, in a large proportion of DFU patients treated with commercial DDM, the skin lesions remained unclosed at 16 weeks (Brigido, 2006), indicating that the efficacy of these DDMs on DFU healing still needs enhancement. To improve this, simultaneously adding exogenous GFs and loading stem cells onto the DDM may be a promising and possible solution to improve the efficiency of DDMs in clinical settings.

Basic fibroblast growth factor (bFGF) is a canonical member of the fibroblast growth factor family, which potently stimulates the proliferation of fibroblasts and capillary endothelial cells that give rise to granulation tissue (Zubair and Ahmad, 2019; Tan et al., 2020). Theoretically, bFGF could be loaded onto the DDM to improve its angiogenic functions. However, bFGF laden on DDMs using conventional approaches, such as hydrogels, nanoparticles, or liposomes (Xiang et al., 2011; Losi et al., 2013; Zhang et al., 2018), always show an inhomogeneous distribution, which may hinder the rapid regeneration of skin tissue. Given that collagen is the main component of DDMs, increasing the collagen binding ability of bFGF may be a good choice for homogeneous bFGF laden on DDMs. Recently, a heptapeptide (TKKTLRT), termed collagen-binding domain (CBD), showed affinity for collagen (Wahyudi et al., 2016), which was fused onto the N-terminus of bFGF to synthesize recombinant bFGF (named CBD–bFGF). CBD–bFGF not only exhibits endothelial inducibility but also can specifically bind collagen and be released in a sustained manner (Hao et al., 2018). Thus, we intended to use CBD–bFGF to improve the angiogenic functions of DDMs.

Apart from the scaffold, the source of seed cells, as well as their loading mode, is another critical factor in tissue engineering (Yin et al., 2013; Font Tellado et al., 2015). Adipose-derived stromal cells (ASCs) are the most commonly used cell source in the field of tissue engineering, owing to their rapid proliferation ability, multi-differentiation potential, and ease of harvest (Li et al., 2018; Luck et al., 2020; Mazini et al., 2020), and they have been widely used in combination with different scaffolds for diabetic wound healing in preclinical and clinical studies. Herein ASCs were selected as the seed cells for constructing tissue-engineered skin grafts. Considering that the cell sheet technique not only achieves highly efficient cell delivery but also preserves cell–cell contact, the secreted ECM and various factors during culture exert a significantly better effect on tissue regeneration than the administration of seed cells *via* suspension or microspheres (Harada et al., 2017). Therefore, we intended to load ASCs on a DDM in the form of a cell sheet. To better load the ASC sheets on the DDM (Chen et al., 2019), a novel book-shaped decellularized dermal matrix (BDDM) would be fabricated, and the ASC sheets would be uniformly sandwiched by the book-shaped scaffold, following a previous study. This mode could enlarge the number of seed cell loadings and facilitate the uniform distribution of seed cells in the scaffold.

In this study, inspired by the “book” appearance, we intended to prepare a DDM with a book-like appearance (termed BDDM). In order to promote the angiogenic inducibility of the BDDM, recombinant bFGF (named as CBD–bFGF) was tethered onto the collagen fibers of BDDM to fabricate a functional scaffold (CBD–bFGF/BDDM). After *in vitro* testing of the bFGF release dynamics and angiogenic functions of CBD–bFGF/BDDM, ASCs were cultured to form a cell sheet and sandwiched by CBD–bFGF/BDDM to construct a tissue-engineered skin graft (ASCs/CBD–bFGF/BDDM). Finally, we tested the ability of ASCs/CBD–bFGF/BDDM to heal full-thickness wounds in a streptozotocin-induced diabetic rat model. This study may provide a promising tissue engineering strategy for DFU healing.

## Materials and Methods

### Ethics Statement

The experimental protocol for the use of Sprague–Dawley (SD) rats in this study was approved by the Animal Ethics Committee of Chenzhou No. 1 People’s Hospital.

### Preparation and Evaluation of the Book-Shaped Decellularized Dermal Matrix

#### Preparation of BDDM

According to the rules on harvesting and processing tissues at Chenzhou No. 1 People’s Hospital, fresh skin tissues were acquired from donors who were undergoing traumatic amputation caused by car accidents. Samples from donors with transmittable diseases, including human immunodeficiency virus, hepatitis, syphilis, and human T-cell lymphotropic virus were excluded. In brief, the skin tissue pieces were incised into rectangular shapes, 2 cm in width and 3 cm in length, and then trimmed into a book shape with three pages (page thickness = 300 μm) (Supplementary Figure 1). After the book-shaped skin samples were rinsed with phosphate buffer solution (PBS), they were rinsed in decellularization solution A (10 mM Tris buffer and 5 mM ethylenediaminetetraacetic acid) for 12 h at room temperature. Then, the epidermal layer was mechanically detached from the dermis and processed within decellularization solution B (1% Triton X-100, 1.5 M potassium chloride, and 50 mM Tris buffer) for 12 h. After that, the samples were digested by 100 μg/ml RNase and 150 IU/ml DNase at 37°C for 2 h, followed by washing with PBS for 12 h. After the samples were lyophilized with a vacuum freeze-drier (FD8-5T, SIM, FL, United States), BDDM was acquired. During decellularization, the aforementioned solutions were supplemented with 1% penicillin–streptomycin–amphotericin B (03-033-1B, BioInd, Israel). Protease inhibitor (one tablet/300 ml, S8820-20TAB, Sigma, United States) was added to all solutions except the nuclease solution. Commercialized DDM was purchased from Beijing Jayyalife Biological Technology Co., Ltd., which has been authorized for clinical application by the Chinese Food and Drug Administration.

#### Evaluating the Histological and Ultrastructural Characteristics in the BDDM

Book-shaped decellularized dermal matrix and natural dermal tissue (NDT) were fixed in 4% neutral paraformaldehyde for 24 h and then embedded in paraffin. After the paraffin-embedded tissue blocks were sectioned at a thickness of 5 μm, the slices were stained with hematoxylin and eosin (H&E), 4′,6-diamidino-2-phenylindole (DAPI), Masson’s trichrome (MT), or anti-fibronectin (Rabbit Anti-Rat, ab268020, Sigma, United States). H&E staining was used together with DAPI to evaluate the elimination of cellular components in BDDM. MT staining combined with anti-fibronectin staining was used to observe the preservation of collagen and fibronectin in the BDDM. To comparatively observe the ultrastructural difference between the BDDM and NDT, the BDDM and NDT were fixed with 0.25% glutaraldehyde solution and sputter-coated with gold for scanning electron microscopy (SEM) (S-3400 N; Hitachi, Japan).

### Preparation of the ASC Sheet

#### ASC Isolation and Identification

After inguinal subcutaneous adipose tissue was harvested from 3-week-old SD rats, the adipose tissues were extensively washed with PBS and then minced aseptically using sterilized surgical scissors, followed by 0.1% type I collagenase digestion (Gibco, United States) for 1.5 h at 37°C in a water bath shaker. Next, complete medium (DMEM/F12 + 10% FBS + 1% antibiotics; Gibco, United States) was added to neutralize the collagenase. After that, the suspension was centrifuged at 1,000 rpm for 5 min, and then the cell aggregate was washed with PBS, resuspended in complete medium, and incubated at 37°C in 5% CO_2_. Upon reaching 70–80% confluence, the cells were passaged. Isolated cells were identified by flow cytometry analysis using antibodies against CD11b, CD29, CD34, CD45, CD90, and CD105 (RAXMX-09011 Kit, Cyagen, United States). *In vitro* osteogenic differentiation, chondrogenic differentiation, and adipogenic differentiation were also used to identify the isolated cells. The passaged three cells were used for further experiments.

#### ASC Sheet Preparation and Evaluation

Adipose-derived stromal cells isolated from SD rats were seeded at 400,000 cells/cm^2^ on temperature-responsive culture dishes (3.5 cm in diameter, UpCell, ThermoFisher Scientific, United States) and cultured in complete medium with 20 μg/ml L-ascorbic acid (Sigma, United States) to form a coherent cell sheet. After spontaneous detachment at room temperature, the ASC sheet was either used to engineer grafts for patching diabetic wounds or histologically evaluated with H&E staining.

### CBD–bFGF/BDDM Fabrication and Evaluation

#### Synthesis of CBD–bFGF

CBD–bFGF was synthesized according to a method described previously in the literature (Zhao et al., 2007; Shi et al., 2017). The CBD–bFGF protein expression vector was transplanted into the BL21 (DE3) strain of *Escherichia coli* and induced by isopropyl β-D-thiogalactopyranoside at 25°C for 8 h. The expressed recombinant protein was purified from the supernatant by pulling down the 6 × His tag. Purified CBD–bFGF was determined by sodium dodecyl sulfate–polyacrylamide gel electrophoresis (SDS-PAGE) and western blotting (Rabbit Anti, ab213204, Abcam, United States). Natural bFGF (NAT-bFGF) expressed in *E. coli* was purchased from PeproTech (100-18B, PeproTech, United States) as a control. The amino acid sequences of CBD–bFGF and NAT-bFGF are listed in Supplementary Table 1.

#### Bioactivity Evaluation of CBD–bFGF

Adipose-derived stromal cells were used to evaluate the activities of CBD–bFGF. For the endothelial inducibility of CBD–bFGF or NAT-bFGF, ASCs were seeded in 12-well plates (Costar) at a density of 10^5^ per well and cultured in complete medium (DMEM/F12 + 10% FBS + 1% antibiotics; Gibco, United States) at 37°C in 5% CO_2_. After 24 h of culture, the medium was changed to DMEM/F12 with 10% FBS with the addition of NAT-bFGF (10 ng/ml) or CBD–bFGF (10 ng/ml). After culturing for 10 days, qRT-PCR was applied to detect the expression of endothelial marker genes (CD31, vWF, and CD144). The primer sequences are listed in Supplementary Table 2. To further assess the endothelial inducibility of CBD–bFGF or NAT-bFGF, CD31 expression in ASCs was evaluated by immunofluorescence assay using an anti-CD31 antibody (ab24590, Abcam, United States). In addition, a Matrigel-based capillary-like tube formation assay was used to evaluate the tube forming capacity of ASCs stimulated by CBD–bFGF or NAT-bFGF. The stimulated ASCs were seeded on a Matrigel (BD Biosciences, United States) substrate and incubated at 37°C in 5% CO_2_ for up to 12 h. The cord-like structures were captured and counted by phase-contrast microscopy. ASCs cultured on tissue culture polystyrene (TCP) without stimulation were used as the control group.

#### CBD–bFGF on BDDM Binding Assay

Considering that the BDDM is full of collagen, we used it as a collagen scaffold to evaluate the binding ability of CBD–bFGF. A reference for the collagen binding assay can be found in published literature (Shi et al., 2017). Briefly, the BDDM (weighing approximately 122.71 ± 1.87 mg) was plated in a 48-well plate and then incubated with gradients of equal molar amounts of NAT-bFGF or CBD–bFGF for 2 h at 37°C. A primary antibody (ab208687, anti-bFGF, Rabbit, Abcam, United States) was added to the plates for 1 h at 37°C. Unbound antibody was removed by three washes with PBS. A secondary antibody (ab97048, goat anti-rabbit IgG H&L, Abcam, United States) was then added, followed by incubation for 1 h at 37°C. After washing with PBS, P-NPP disodium (2 mg/ml) was added for 10 min at room temperature, followed by termination of the reaction by the addition of an equal volume of 0.2 M NaOH. The absorbance was measured at 405 nm using a plate reader.

#### Controlled Release Assay of CBD–bFGF From CBD–bFGF/BDDM *in vitro*

After the BDDM (weighing approximately 120.23 ± 0.37 mg) was loaded with 1 μM NAT-bFGF or CBD–bFGF, it was suspended in 500 μl of PBS with 1% antibiotics at 37°C under continuous shaking. The PBS was collected and changed every 24 h. At each time point from day 0 to day 14, the concentration of bFGF in solution was analyzed by a human bFGF ELISA kit (70-EK1119-96, MultiSciences, China).

#### Cell Viability and Proliferation

Adipose-derived stromal cells (1.2 × 10^4^/cm^2^, passage 3) were seeded onto CBD–bFGF/BDDM or T (as a control group). After 3 days of culture, a live/dead assay kit (40747ES76, Yeasen, Shanghai, China) was used to stain the ASCs cultured on CBD–bFGF/BDDM or TCPs. Images of the green-stained (live) cells and red-stained (dead) cells were captured by fluorescence microscopy with an excitation wavelength of 488/594 nm. Cell viability was calculated as follows: (live cells/total cells) × 100%. Additionally, a CCK8 kit (70-CCK8100, MultiSciences, China) was used to assess ASC proliferation on CBD–bFGF/BDDM on post-seeding days 1, 3, 5, 7, 9, and 11.

#### *In vitro* Endothelial Inducibility of CBD–bFGF/BDDM

The endothelial differentiation of ASCs induced by CBD–bFGF/BDDM, NAT-bFGF/BDDM, and TCPs was evaluated using 24-well Transwell inserts with 8-μm-pore-size filters (Costar 3422, Corning, United States) (*n* = 4). Briefly, CBD–bFGF/BDDM or NAT-bFGF/BDDM was transferred to the upper chamber. An upper chamber without a scaffold was set as a control group (TCPs). ASCs (5 × 104 cells/well, passage 3) were seeded into the lower chamber and cultured in complete medium (DMEM/F12 + 10% FBS + 1% antibiotics, Gibco, United States) at 37°C in 5% CO_2_. After culturing for 10 days, qRT-PCR was applied to detect the expression of endothelial marker genes (CD31, vWF, and CD144). The primer sequences are listed in Supplementary Table 2. To further assess the endothelial inducibility of CBD–bFGF/BDDM or NAT-bFGF/BDDM, CD31 expression in ASCs was evaluated with an immunofluorescence assay using an anti-CD31 antibody (ab182981, Abcam, United States). Additionally, a Matrigel-based capillary-like tube formation assay was used to evaluate the tube forming capacity of ASCs co-cultured with CBD–bFGF/BDDM or NAT-bFGF/BDDM. The co-cultured ASCs were seeded on a Matrigel (BD Biosciences, United States) substrate and incubated at 37°C in 5% CO_2_ for up to 12 h. The cord-like structures were captured and counted by phase-contrast microscopy.

#### Immunogenicity of CBD–bFGF/BDDM

The low immunogenicity of CBD–bFGF/BDDM was determined using 24-well Transwell inserts with 8-μm-pore-size filters (Costar 3422, Corning, United States) (*n* = 3). Briefly, RAW 264.7 cells at a density of 5.0 × 104 cells/well were cultured with complete medium (high-glucose DMEM + 10% FBS + 1% antibiotics; Gibco, United States) in the lower compartment. In the upper compartment, only complete medium was used as a negative control (TCP), while complete medium with 10 μg/ml lipopolysaccharide (LPS) was used as a positive control. Complete medium with CBD–bFGF/BDDM was set as the CBD–bFGF/BDDM group. After 7 days of culture, we collected the culture supernatant to detect TNF-α, IL-6, and IL-1β levels using ELISA kits (Multi Sciences, China).

### *In vivo* Assessment in a Diabetic Rat Model

#### Tissue-Engineered Skin Graft Construction

Based on the saturation binding amount of CBD–bFGF on the BDDM, we immersed the BDDM in CBD–bFGF solution (1 μM), followed by incubation at 4°C for 12 h to acquire CBD–bFGF/BDDM. Additionally, two slices of ASC sheets were inserted into the page gaps of CBD–bFGF/BDDM, and then one ASC sheet slice was used to cover the button of CBD–bFGF/BDDM. Hence, a tissue-engineered skin graft was constructed (termed ASCs/CBD–bFGF/BDDM).

#### The Streptozotocin-Induced Diabetic Rat Model

In order to acquire diabetic rats, a single dose (75 mg/kg) of streptozotocin solution was injected into the abdominal cavity of adult male SD rats (weight: 200–250 g). After that, an electronic glucometer was used to measure the blood glucose levels on days 0, 3, and 7. Animals with blood glucose levels higher than 15 mM and that showed weight loss, polyuria, and polydipsia were considered diabetic and prepared for *in vivo* wound healing experiments. At 1 week later, the rats were anesthetized with pentobarbital (3%, 1.2 ml/kg) and then shaved with an electric clipper to clear the hair on the dorsal surface. Then, the dorsal surface of the animal was disinfected with 5% povidone–iodine solution, and a rounded full-thickness wound with a diameter of 20 mm was produced on the dorsal surface of the diabetic rats. The wounds of these operated rats were patched with four different tissue-engineered skin grafts: commercial DDM (DDM group), BDDM (BDDM group), CBD–bFGF/BDDM (CBD–bFGF/BDDM group), or ASCs/CBD–bFGF/BDDM (ASCs/CBD–bFGF/BDDM group). The implanted grafts were sutured into the wound site using black silk suture (SK526; Teleflex MedicalOEM, South Korea).

#### Wound Healing Assay

On postoperative days 0, 7, 14, and 21, the skin wounds of each rat were photographed, and these images were imported into Image-Pro Plus (version 6.0.0; Media Cybernetics Inc.) for calculation of wound closure by two independent investigators (Xin Shi and Xin Zhao). The percentage of wound closure was calculated as follows: (A0−At)/A0 × 100%, where “A0” indicates the area of the original wound, and “At” is the area of the wound at the measured timepoint.

#### Histological Evaluation

The rats were sacrificed 14 and 21 days after surgery (eight animals per group per time point) to harvest tissue samples by excising the graft together with a surrounding rim of normal skin. After fixation in 4% neutral paraformaldehyde for 24 h, the samples were dehydrated in ethanol, cleared in xylene, and then embedded in paraffin. The paraffin-embedded samples were sectioned at a thickness of 5 μm and then stained with H&E and MT for histological evaluation, including wound re-epithelization and collagen deposition. In the H&E-stained sections, the percentage of re-epithelialization (*E*%) was calculated according to previously described methods: *E*% = Wt/Wo × 100, where “Wo” is the length of the original wound, and “Wt” is the length of the neo-epithelium across the surface of the wound. In the MT-stained sections, the degree of collagen maturity in the wound beds was calculated by detecting the mean staining intensity for Masson (three random visual fields per section) using Image-Pro Plus 6 software.

#### Angiogenesis Evaluation

On postoperative day 7, wound samples from each group were used to detect whether angiogenesis had occurred in the granulation tissue of the wound bed. Briefly, the wound samples were sectioned and incubated with mouse anti-CD31 antibody (ab24590, Abcam, United States) and rabbit anti-αSMA (ab124964, Abcam, United States) at 4°C overnight, and then the sections were incubated with goat anti-mouse IgG H&L secondary antibody (ab150116, Abcam, United States) and goat anti-rabbit IgG H&L secondary antibody (ab150077, Abcam, United States) for 90 min at room temperature. The nuclei were stained with DAPI (Sigma Aldrich, no. D9542, United States). Images were captured with a fluorescence microscope (ApoTome 2, Carl Zeiss, Germany). The numbers of blood vessels in the four groups were determined by counting in three random fields/section between the wound edges using Image-Pro Plus 6.

### Statistical Analysis

All data are expressed as mean ± standard deviation. We used Student’s *t*-test for the comparison between two groups and one-way ANOVA with a *post hoc* test for the comparison above two groups. *P* < 0.05 was considered statistically significant. All the analyses were performed using SPSS 25.0 software (SPSS, United States).

## Results

### Identification of ASCs and Morphology of the ASC Sheet

#### Isolation and Identification of ASCs

**FIGURE 1:**
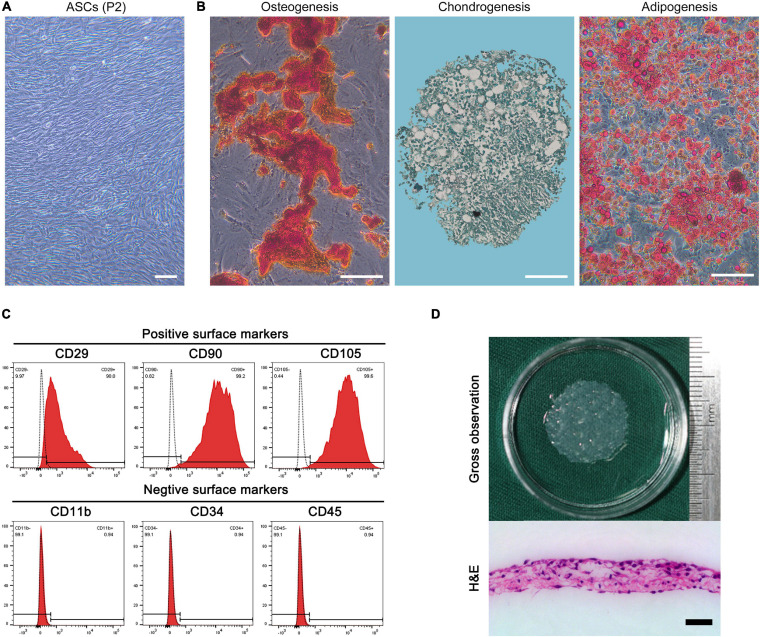
**(A)** Morphology of rat adipose-derived stromal cells (ASCs) at P2. Bar = 100 μm. **(B)** Osteogenic differentiation, chondrogenic differentiation, and adipogenic differentiation of ASCs revealed by alizarin red, alcian blue, and oil red O staining. Bar = 100 μm. **(C)** Flow cytometry analysis of the expression of cell surface markers related to stem cell phenotype in ASCs, which were positive for CD29, CD90, and CD105 and negative for CD11b, CD34, and CD45. **(D)** Macroscopic morphology and H&E-stained image of the ASC sheet. Bar = 50 μm.

The isolated cells in the culture plate showed a spindle-shaped morphology under a phase-contrast microscope (Figure 1A). Under osteogenic, chondrogenic, or adipogenic induction, the isolated cells were differentiated into osteocytes, chondrocytes, or adipocytes as shown by positive staining for alizarin red, alcian blue, and oil red O, respectively (Figure 1B). As determined by flow cytometry analysis, these isolated cells were positive for CD29, CD90, and CD105 but negative for CD11b, CD34, and CD45 (Figure 1C).

#### Morphology of the ASC Sheet

The ASC sheet presented a homogeneous membrane structure, was composed of a large number of cells, and secreted ECM in the H&E-stained image (Figure 1D).

### Characteristics of the CBD–bFGF/BDDM

#### Gross Observations and Morphology of the BDDM

**FIGURE 2:**
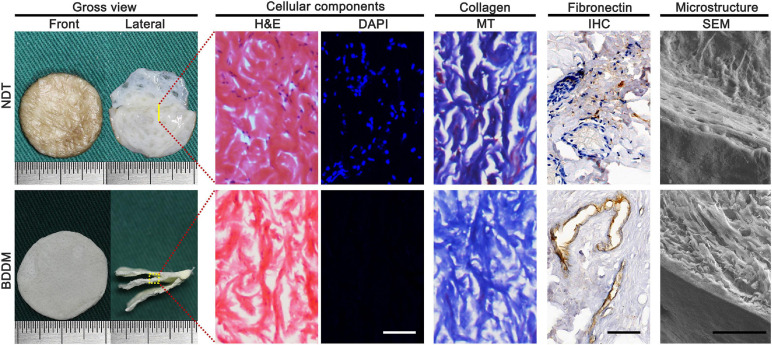
Gross observation, cellular components, collagen and fibronectin preservation, the microstructure of the native dermal matrix, and book-shaped decellularized dermal matrix. Bar = 100 μm.

As shown in Figure 2, the lyophilized BDDM presented a book-shaped appearance with three pages, each approximately 300 μm thick. Histologically, H&E- and DAPI-stained sections of the BDDM showed that all the cellular content had been clearly removed from the dermis. MT staining and immunohistochemistry staining using anti-fibronectin determined that our decellularization process did not deteriorate the structural integrity of the normal dermis but completely removed the epidermal layer-affiliated hair ducts and sweat glands. The SEM images showed a well-preserved NDT microstructure of the BDDM.

#### CBD–bFGF Has a Similar Bioactivity to That of NAT-bFGF

**FIGURE 3:**
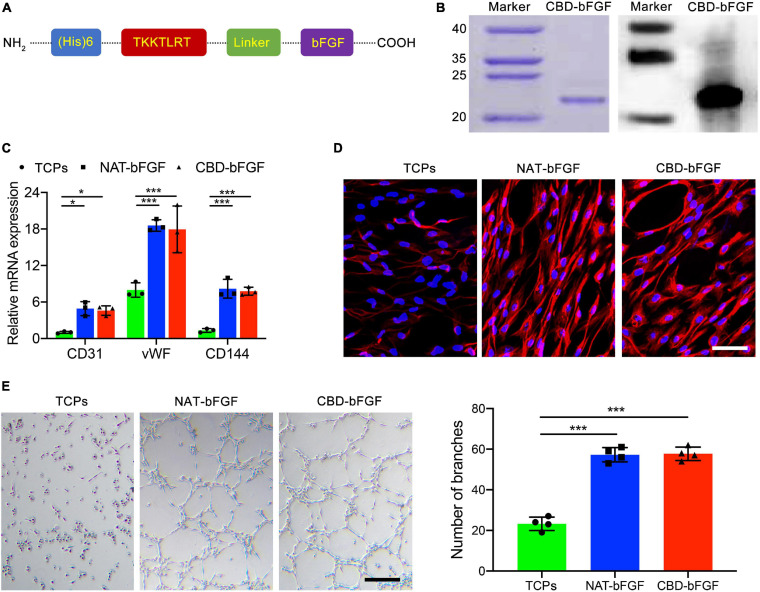
**(A)** Schematic illustration of the full chemical structure of the collagen-binding domain–basic fibroblast growth factor (CBD–bFGF). **(B)** CBD–bFGF was identified by SDS-PAGE together with western blotting. **(C)** The expression of CD31, vWF, and CD144 in the adipose-derived stromal cells (ASCs) after having been induced by CBD–bFGF or natural (NAT)-bFGF for 10 days as evaluated using qRT-PCR. **(D)** The expression of CD31 in the ASCs after culturing with CBD–bFGF or NAT-bFGF for 10 days, stained with anti-CD31 antibody. Bar = 50 μm. **(E)** Phase-contrast photomicrographs of the differentiated ASCs subsequently plated onto Matrigel for 12 h demonstrate more formation of capillary-like structures under the stimulation of CBD–bFGF and NAT-bFGF for 10 days. Bar = 100 μm. Data are shown as means ± standard deviation (*P < 0.05, **P < 0.01, and ***P < 0.001).

Collagen-binding domain was fused to the N-terminus of bFGF with a linker sequence (Figure 3A). CBD–bFGF was identified by SDS-PAGE and western blotting (Figure 3B). The endothelial differentiation of ASCs was measured by evaluating CD31, vWF, and CD144 mRNA transcript levels using qRT-PCR. After 10 days of culture, ASCs (*n* = 4) in the NAT-bFGF group or CBD–bFGF group expressed significantly higher endothelial cell-specific markers (CD 31, vWF, and CD144) than those in the TCP group (Figure 3C). Furthermore, immunofluorescence staining assays confirmed the protein expression of CD31 in ASCs after NAT-bFGF or CBD–bFGF induction (Figure 3D). In addition, more capillary-like structures formed when the ASCs were plated onto Matrigel after induction by NAT-bFGF or CBD–bFGF (Figure 3E).

#### Collagen Binding Ability of CBD–bFGF to BDDM

**FIGURE 4:**
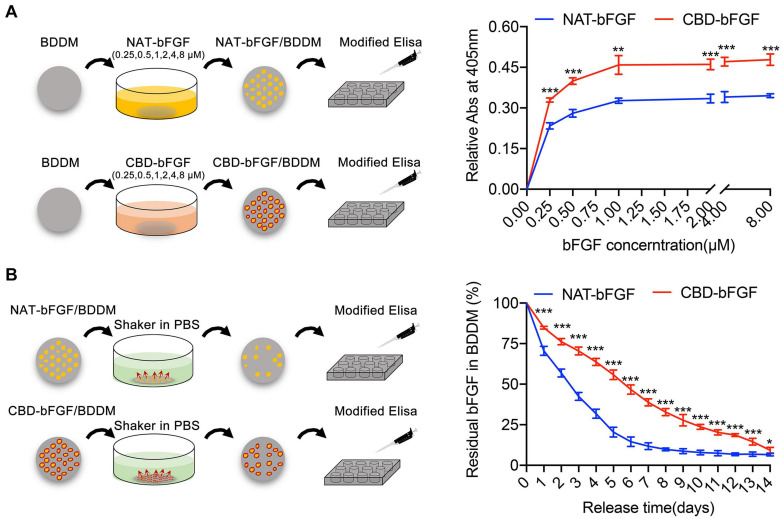
**(A)** The binding curves of collagen-binding domain–basic fibroblast growth factor (CBD–bFGF) or natural (NAT)-bFGF on the collagens of book-shaped decellularized dermal matrix (BDDM). **(B)** The curves showed the residual proportions of CBD–bFGF or NAT-bFGF in CBD–bFGF/BDDM or NAT-bFGF/BDDM after rinsing in phosphate-buffered solution every day. Data (*n* = 4) are shown as means ± standard deviation (*P < 0.05, **P < 0.01, and ***P < 0.001).

As shown in Figure 4A, CBD–bFGF showed a stronger binding ability to the BDDM than NAT-bFGF in a dose-dependent manner. At lower concentrations, similar amounts of NAT-bFGF and CBD–bFGF were bound to the collagen of the BDDM, indicating that NAT-bFGF possesses some collagen binding ability, which was demonstrated by other studies (Kanematsu et al., 2004; Miyoshi et al., 2005). At a concentration of 1.0 μM, the binding of NAT-bFGF and CBD–bFGF nearly reached their maximum. At concentrations of 0.25, 0.5, 1.0, 2.0, 4.0, and 8.0 μM, the amount of CBD–bFGF bound on the BDDM was significantly higher than the amount of NAT-bFGF bound. These results indicated that the fused CBD significantly increased the collagen binding ability of bFGF.

#### Sustained Release of CBD–bFGF From CBD–bFGF/BDDM

As shown in Figure 4B, the sustained release of CBD–bFGF or NAT-bFGF was evaluated over 14 days after binding with the BDDM. Our results determined that NAT-bFGF was rapidly released from the BDDM, while CBD–bFGF was slowly released from the BDDM. These results indicated that CBD–bFGF can specifically tether BDDM, resulting in a more sustained release from BDDM than NAT-bFGF.

#### Endothelial Inducibility of CBD–bFGF/BDDM *in vitro*

**FIGURE 5:**
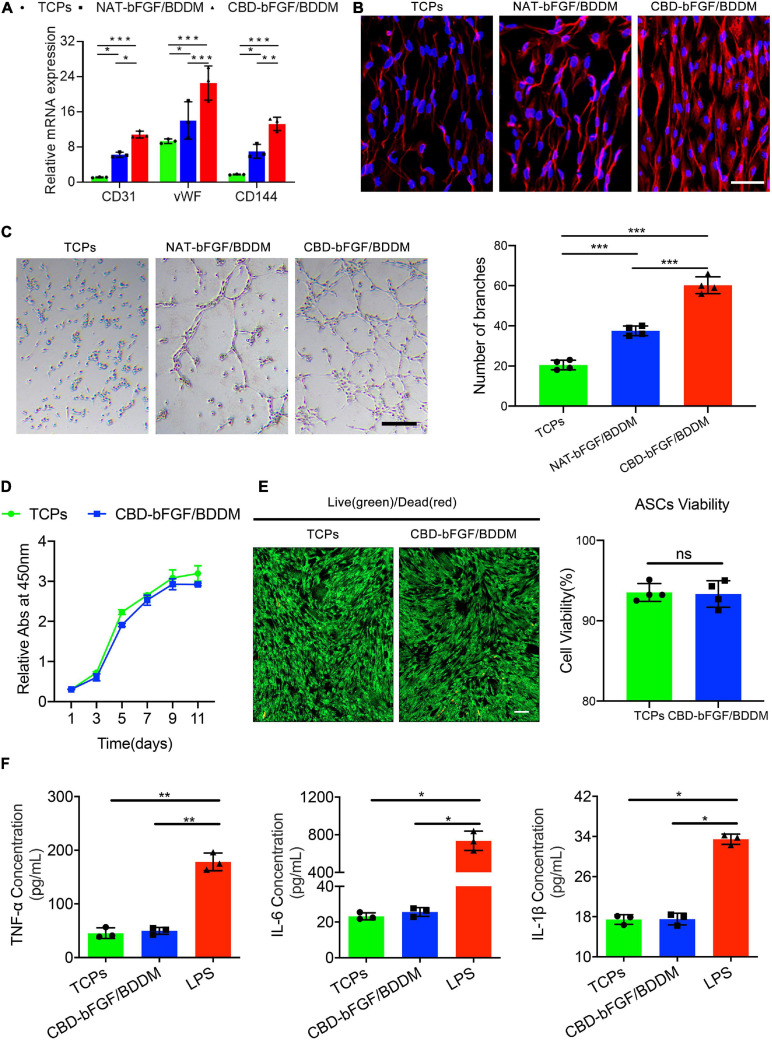
**(A)** Endothelial cell-specific markers’ (CD31, vWF, and CD144) expression quantified by qRT-PCR demonstrates that natural (NAT)–basic fibroblast growth factor (bFGF)/book-shaped decellularized dermal matrix (BDDM) or collagen-binding domain (CBD)–bFGF/BDDM induced the expression of these endothelial molecular markers. **(B)** Immunofluorescence staining assays for CD31 expression in the adipose-derived stromal cells (ASCs) cultured with tissue culture polystyrenes (TCPs), NAT-bFGF/BDDM, or CBD–bFGF/BDDM for 10 days. Bar = 50 μm. **(C)** Phase-contrast photographs of the differentiated ASCs subsequently plated onto Matrigel for 12 h show more formation of capillary-like structures under the stimulation of CBD–bFGF/BDDM for 10 days. Bar = 100 μm. **(D)** Comparative cell proliferation assay of ASCs seeded on the TCPs and CBD–bFGF/BDDM using CCK8 assay (*n* = 4). **(E)** Representative photographs show the live (green) and dead (red) ASCs after culturing with ASCs for 3 days and the viability analysis for the cells on the TCPs and CBD–bFGF/BDDM. Bar = 100 μm. **(F)** Pro-inflammatory cytokine (TNF-α, IL-6, and IL-1β) secreted by RAW 264.7 in the supernatant of TCPs, CBD–bFGF/BDDM, or lipopolysaccharide group. Data are shown as means ± standard deviation (*P < 0.05, **P < 0.01, and ***P < 0.001).

After the ASCs were cultured with TCPs, NAT-bFGF/BDDM, or CBD–bFGF/BDDM for 10 days, the ASCs cultured with CBD–bFGF/BDDM expressed the highest mRNA for endothelial cell-specific genes (CD31, vWF, and CD144) among the three groups, while the ASCs cultured with NAT-bFGF/BDDM also showed increased mRNA levels of CD31, vWF, and CD144 (Figure 5A). Immunofluorescence assays further validated that the level of the endothelial marker CD31 was upregulated in ASCs cultured with CBD–bFGF/BDDM, indicating that these cells successfully differentiated into endothelial cells (Figure 5B). In addition, a Matrigel tube formation assay was used to evaluate the ability of the ASCs to form capillaries (Figure 5C). Our results indicated that the ASCs cultured with CBD–bFGF/BDDM had tube formation ability.

#### ASC Proliferation and Viability on CBD–bFGF/BDDM

As shown in Figure 5D, the ASCs seeded on CBD–bFGF/BDDM showed increased proliferation compared with the ASCs cultured on TCPs using a CCK8 assay. Additionally, using a live/dead assay, most of the ASCs on CBD–bFGF/BDDM displayed green fluorescence (live cells) after 3 days of culture, while a few ASCs displayed red fluorescence (dead cells). Statistically, the viability of ASCs on CBD–bFGF/BDDM was lower than that on TCPs, but the difference was not significant (Figure 5E).

#### Immunogenicity of CBD–bFGF/BDDM

After RAW 264.7 cells were cultured with TCPs, CBD–bFGF/BDDM, and LPS, the supernatants of the TCP and CBD–bFGF/BDDM groups showed similar levels of pro-inflammatory cytokines (TNF-α, IL-6, and IL-1β), and both of these groups showed significantly lower expression levels than those in the LPS group (Figure 5F). These results indicated that CBD–bFGF/BDDM is a biomaterial with low immunogenicity.

### *In vivo* Performance of ASCs/CBD–bFGF/BDDM on Diabetic Wound Healing

#### Morphology of ASCs/CBD–bFGF/BDDM

**FIGURE 6:**
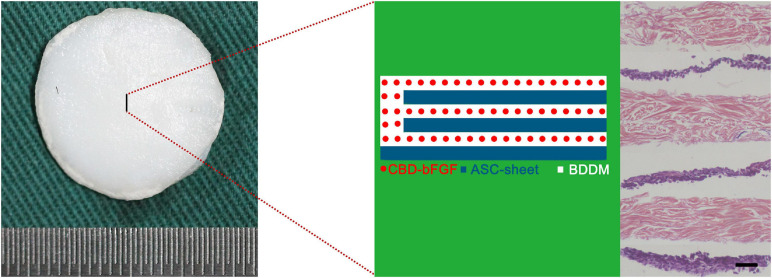
Gross appearance and histological morphology of adipose-derived stromal cells/collagen-binding domain–basic fibroblast growth factor/book-shaped decellularized dermal matrix. Bar = 100 μm.

CBD–bFGF/BDDM showed a book-like appearance with three pages. For ASCs/CBD–bFGF/BDDM (Figure 6), three slices of ASC sheets were interleaved into the page gaps of CBD–bFGF/BDDM. Histologically, the ASC sheet was composed of a large number of ASCs that secreted ECM.

#### ASCs/CBD–bFGF/BDDM Accelerate Diabetic Wound Healing

**FIGURE 7:**
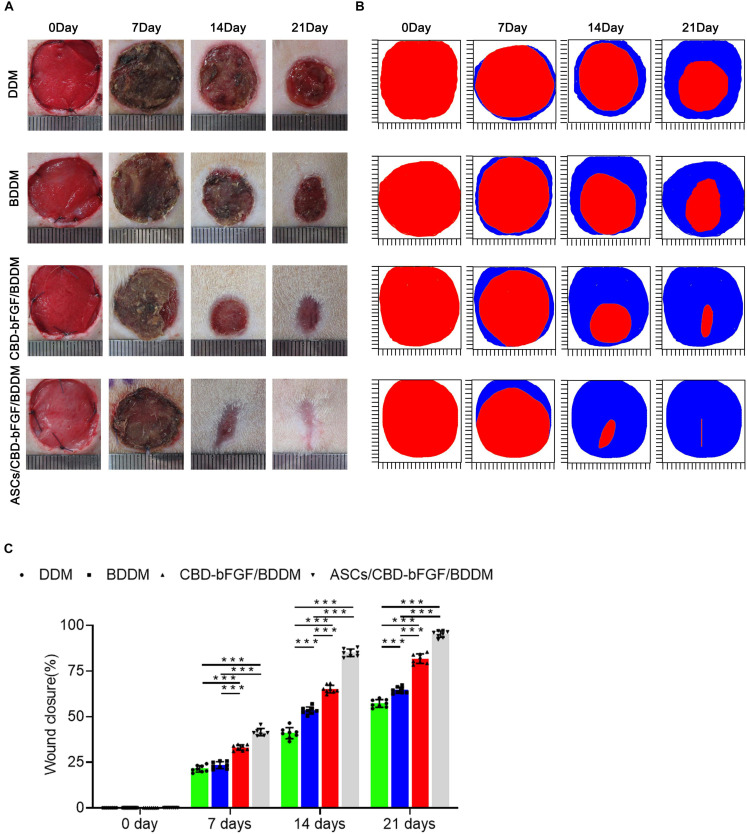
**(A)** Overview of the size change of the wounds made in the dorsal skin of diabetic mice among the four groups at postoperative days 0, 7, 14, and 21. **(B)** Traces of wound bed closure for each treatment group *in vivo*. The red area indicates the wound area at day 0, and the blue area indicates the wound area at postoperative days 7, 14, and 21. **(C)** Statistical analysis of the wound area in each group at postoperative days 0, 7, 14, and 21. Data are shown as means ± standard deviation (*P < 0.05, **P < 0.01, and ***P < 0.001).

**FIGURE 8:**
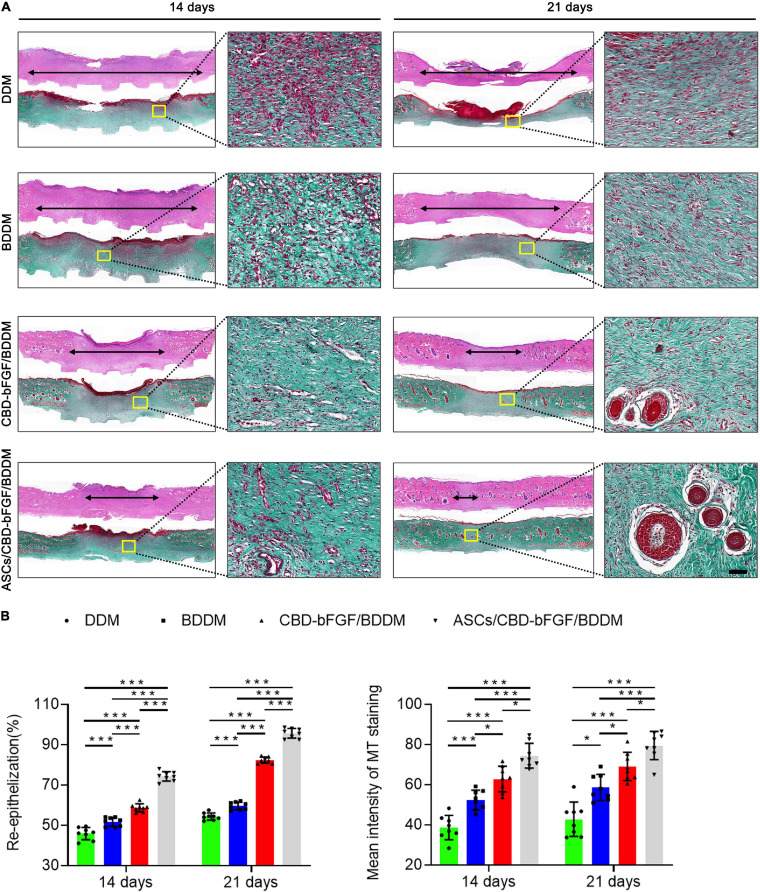
**(A)** H&E and Masson’s trichrome (MT) staining of wound sections in the decellularized dermal matrix (DDM), book-shaped decellularized dermal matrix (BDDM), collagen-binding domain–basic fibroblast growth factor (CBD–bFGF)/BDDM, or adipose-derived stromal cells (ASCs)/CBD–bFGF/BDDM groups at 14 or 21 days after the operation. The double-headed black arrows indicate the edges of the scars. Bar = 100 μm. **(B)** Quantitative analysis of the re-epithelialization extent and the mean intensity of MT staining in the histological sections of the DDM, BDDM, CBD–bFGF/BDDM, or ASCs/CBD–bFGF/BDDM groups. Data are shown as means ± standard deviation (*P < 0.05, **P < 0.01, and ***P < 0.001).

In order to evaluate the *in vivo* performance of ASCs/CBD–bFGF/BDDM on diabetic wound healing, we covered the full-thickness skin incisions created on the back of diabetic rats with the prepared materials (DDM, BDDM, CBD–bFGF/BDDM, and ASCs/CBD–bFGF/BDDM). As shown in Figure 7, no sign of inflammation or infection formation was observed in the material-covered wounds at different time points. New epidermis grew from the edge of the skin wound and gradually extended to the center, resulting in a reduction in the wound area. After 21 days, the skin wounds in the ASCs/CBD–bFGF/BDDM group were almost healed, while there were obvious wounds in the DDM, BDDM, or CBD–bFGF/BDDM groups. At 7 days after transplantation, the wound closure rates in the DDM and BDDM groups were similar without a significant difference, but both were significantly lower than those of the CBD–bFGF/BDDM and ASCs/CBD–bFGF/BDDM groups. The wound closure rate in the ASCs/CBD–bFGF/BDDM group presented a significantly higher value than that of the CBD–bFGF/BDDM group. At 14 days after transplantation, the ASCs/CBD–bFGF/BDDM group showed a significantly increased wound closure rate compared with the DDM, BDDM, and CBD–bFGF/BDDM groups. Histologically, H&E staining was used to evaluate the extent of re-epithelialization of the wound defects. As shown in Figure 8, the double-headed arrows indicate the length without re-epithelialization in the wound. The rate of re-epithelialization in the ASCs/CBD–bFGF/BDDM group was the highest among the four groups at postoperative days 14 and 21, and the difference was statistically significant. Additionally, MT staining and quantitative analysis were used to evaluate collagen deposition and maturation. As shown in Figure 8, on postoperative day 14, immature collagen could be seen surrounding the skin fibroblasts in the DDM and BDDM groups, while extensive deposition of collagen fibers was observed in the wound beds of the CBD–bFGF/BDDM and ASCs/CBD–bFGF/BDDM groups. Moreover, on postoperative day 21, there were more collagen deposition and thick wavy collagen fibers in the CBD–bFGF/BDDM and ASCs/CBD–bFGF/BDDM groups than in the DDM and BDDM groups, in which the collagen fibers showed an ordered arrangement pattern with the regeneration of some hair follicles. Quantitative analysis revealed that the collagen contents in the CBD–bFGF/BDDM and ASCs/CBD–bFGF/BDDM groups were significantly higher than those in the DDM and BDDM groups, and the collagen content in the ASCs/CBD–bFGF/BDDM group was significantly higher than that of the CBD–bFGF/BDDM group. These data indicated that ASCs/CBD–bFGF/BDDM could efficiently promote the repair of diabetic wounds.

#### ASCs/CBD–bFGF/BDDM Stimulates Angiogenesis in Diabetic Wounds

**FIGURE 9:**
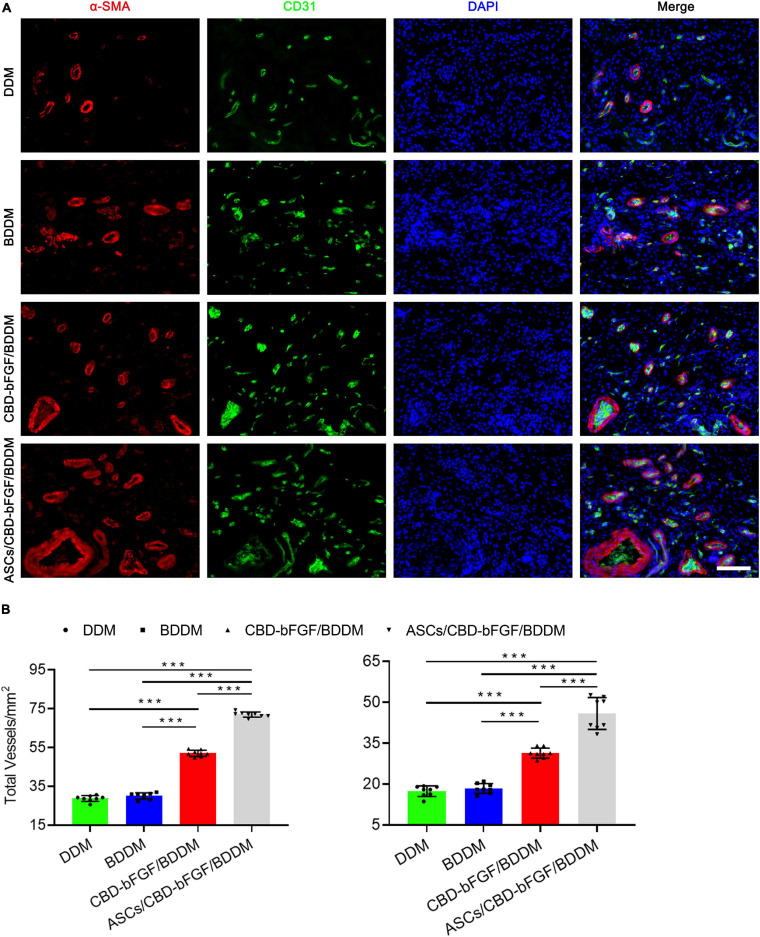
**(A)** Double immunofluorescent staining for CD31 and αSMA in wounds covered with decellularized dermal matrix, book-shaped decellularized dermal matrix (BDDM), collagen-binding domain (CBD)–basic fibroblast growth factor (bFGF)/BDDM, adipose-derived stromal cells/CBD–bFGF/BDDM at day 7 post-wounding. Bar = 50 μm. **(B)** Quantitative analysis of the number of total blood vessels and mature blood vessels in wounds at day 7 post-wounding. Data are shown as means ± standard deviation (*P < 0.05, **P < 0.01, and ***P < 0.001).

Considering that CBD–bFGF/BDDM showed superior endothelial inducibility *in vitro*, we evaluated new blood vessel formation at the wound site at day 7 post-wounding to explore the efficacy of ASCs/CBD–bFGF/BDDM on the stimulation of angiogenesis. Thus, the total number of blood vessels and the number of mature blood vessels in the wound sites covered with DDM, BDDM, CBD–bFGF/BDDM, and ASCs/CBD–bFGF/BDDM were identified by CD31 immunostaining and double-staining for CD31 and αSMA, respectively (Figure 9A), from which the average densities of the total and mature vessels were quantified (Figure 9B). Our results showed that CBD–bFGF/BDDM- or ASC/CBD–bFGF/BDDM-covered wounds significantly enhanced the number of total and mature blood vessels compared to DDM- or BDDM-covered wounds, and significantly more vessels were detected in the ASC/CBD–bFGF/BDDM-covered wounds than in the CBD–bFGF/BDDM-covered wounds.

## Discussion

**FIGURE 10:**
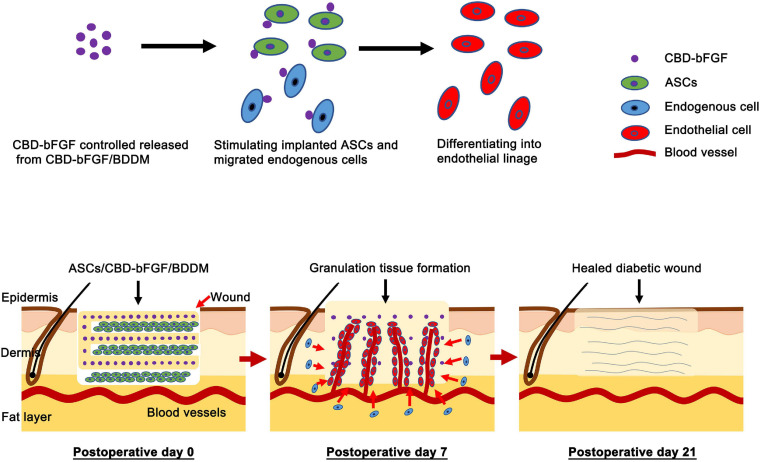
Schematic illustrating the application of adipose-derived stromal cells (ASCs)/collagen-binding domain–basic fibroblast growth factor (CBD–bFGF)/book-shaped decellularized dermal matrix (BDDM) in diabetic wound healing and their potential mechanisms. The CBD–bFGF/BDDM releases the tethered bFGF in a controlled pattern and then stimulates the implanted ASCs, and the endogenous cells migrated into the wound site down into the endothelial lineage, thus promoting diabetic wound healing, including enhancing the angiogenesis, granulation tissue formation, collagen deposition, and re-epithelialization.

Several kinds of commercialized DDMs have been authorized to patch DFUs in the clinic (Guo et al., 2017; Zelen et al., 2018). However, for a large proportion of DFU patients treated with these commercialized DDMs, their skin lesions remain unclosed at 16 weeks (Brigido, 2006), indicating that the efficacy of these DDMs on DFU healing still needs enhancement. To improve the efficiency of DDMs, BDDM, which is highly biomimetic to native dermal tissue in its histology, microstructure, and ingredients, was fabricated. The *in vitro* results indicated that BDDM has no biotoxicity and low immunogenicity and is suitable for ASC attachment and proliferation. Then, an engineered bFGF capable of binding collagen was synthesized and tethered onto the collagen of BDDM to fabricate CBD–bFGF/BDDM with superior endothelial inducibility. Lastly, an ASC sheet was interleaved into the page gaps of CBD–bFGF/BDDM to construct a tissue-engineered graft (ASCs/CBD–bFGF/BDDM). The *in vivo* performance of ASCs/CBD–bFGF/BDDM or CBD–bFGF/BDDM on DFU healing was elucidated using a diabetic rat model. The *in vivo* results indicated that ASCs/CBD–bFGF/BDDM can release tethered bFGF, stimulate laden ASCs down into the entheogenic lineage, and consequently improve angiogenic responses in diabetic wound sites, eventually enhancing the number of blood vessels in the wounds (Figure 10). This study provides a new covering graft for the treatment of DFUs.

Generally, skin wound healing is characterized by a series of sequential stages, including inflammation, proliferation, and remodeling, which involve multiple cells, growth factors, and extracellular signals (Martin, 1997). In the case of diabetes, skin wounds usually occur in combination with continuous inflammation, cell dysfunction, and limited angiogenesis, leading to delayed wound healing (Cho et al., 2019). Conventional treatments for DFUs include pressure offloading, surgical debridement, infection management using antibiotics, and surgical treatment to restore blood flow to wounds (Brem et al., 2004; Cavanagh et al., 2005). However, these treatments are mainly focused on how to close the wounds instead of addressing the underlying pathophysiology of the wounds; thus, their efficacy in improving DFU healing is limited, resulting in the frequent recurrence of wounds and even amputation. To address these shortcomings, various cell-based therapies have been investigated to enhance the healing of DFUs (Kirby et al., 2015; Cho et al., 2019), in which the transplanted cells may replace the resident cells in the diabetic wound and contribute to immune modulation, cell differentiation, angiogenesis, ECM production, growth factor production, and wound contraction (Kirby et al., 2015). In cell-based strategies for wound healing, stem cells are the most commonly used cell source. Currently, stem cells isolated from adipose tissue (ASCs), bone marrow (BMSCs), or umbilical cord blood (UCB-MSCs) have witnessed success in accelerating diabetic wound healing, with the enhancement of wound closure and microvessel density (Luo et al., 2010; Lu et al., 2011; Kuo et al., 2016; Shi et al., 2016). Among these types of stem cells, ASCs are relatively abundant and easily accessible (Kirby et al., 2015; Cho et al., 2019), making them the most suitable and convenient for clinical transplantation. However, the mode of ASC transplantation, such as *in situ* injection or intravenous injection, leads to ASC loss and uneven distribution, which cannot realize the on-site local delivery of abundant ASCs, thus compromising the efficacy and functions of ASCs (Sukho et al., 2017). To maximize the therapeutic effects of ASC transplantation, we used a temperature-responsive culture dish supplemented with ascorbic acid in the culture medium to create ASC sheet and then developed CBD–bFGF/BDDM to sandwich these ASC sheets. The ASC sheets showed good preservation of the cell–cell interactions within the multicellular aggregates along with secreted factors, which provide protection from the harsh chronic wound microenvironment, thus increasing the survival rate of transplanted cells (Yang et al., 2005; Neo et al., 2016). In addition, this CBD–bFGF/BDDM provides a temporary residence for maintaining the viability and activating the angiogenic functions of ASCs.

Apart from the mode of cell transplantation, the *in situ* environment also plays a critical role in the therapeutic effects of transplanted cells. In diabetic wounds, the chronic pro-inflammatory state with the constant infiltration of neutrophils and macrophages disrupts the balance of cytokine and growth factor production, which can directly impair the transplanted cells responsible for angiogenesis, ECM deposition, and granulation tissue formation (Gurtner et al., 2008; Eming et al., 2014). Among them, neovascularization and sufficient blood flow are critical for the process of wound healing since they function in the delivery of oxygen and nutrients into the healing site and create an accelerating effect for subsequent granulation tissue formation (Ren et al., 2018). Considering that ASCs have the potential to enter endothelial lineage under the stimulation of bFGF (Ning et al., 2009; Khan et al., 2017), CBD–bFGF/BDDM, which is capable of sustained bFGF release, was developed for sandwiching the ASC sheets in our study, which can yield the homogeneous distribution and alignment of a large number of ASCs and support their differentiation toward endothelial lineage. As shown in Figure 2, no detectable cellular components were present in the BDDM. Histological images also showed that the BDDM was highly biomimetic to NDT in terms of its histology, microstructure, and ingredients. In addition, the *in vitro* results showed that CBD–bFGF/BDDM has the ability to sustain the release of bFGF, thus providing a microenvironment to stimulate ASCs to differentiate into endothelial lineages. In our study, sectioning NDT into a book shape before decellularization could enlarge the tissue surface area for acellular reagent permeation, thus achieving rapid decellularization while preserving the morphological and compositional properties of the dermis. Moreover, the page gaps in the prepared BDDM could provide a physical channel facilitating the ASC sheet laden into the scaffold homogeneously, thus exposing most of the sandwiched ASCs to bFGF stimulation. This is one of the reasons why the diabetic rats in the ASCs/CBD–bFGF/BDDM group showed significantly better angiogenesis than the rats in the other groups. In addition, the implanted ASC sheets can release bioactive substances in a paracrine manner, including cytokines, growth factors, signaling lipids, and nucleic acids (Torres-Torrillas et al., 2019; Câmara et al., 2020), to optimize the local immune environment at the healing interface and promote angiogenesis and stromal cell migration. As a result, more granulation tissue formed at the healing site.

Using a streptozotocin-induced diabetic rat model, we found that the speed of skin wound closure increased gradually and significantly in the DDM, BDDM, CBD–bFGF/BDDM, and ASCs/CBD–bFGF/BDDM groups. Histologically, the wound re-epithelialization and collagen contents presented an increasing pattern similar to that of wound closure among the four groups. The reasons for this difference may be that (1) skin wound healing in the four groups undergoes a complex process, which successively includes endogenous cell infiltration, scaffold internalization and vascularization, and the formation of granulation tissues. The page gaps of BDDM, CBD–bFGF/BDDM, and ASCs/CBD–bFGF/BDDM could provide a physical channel that facilitates the migration of endogenous cells into the graft, thus accelerating scaffold internalization and vascularization. This may be the main reason why the skin wounds in the BDDM, CBD–bFGF/BDDM, and ASCs/CBD–bFGF/BDDM groups showed significantly better repair than those in the DDM group; (2) compared with the BDDM, CBD–bFGF/BDDM, and ASCs/CBD–bFGF/BDDM were capable of releasing tethered bFGF in a controlled manner. The migrated endogenous cells, especially MSCs, endothelial cells, and fibroblasts, were continuously stimulated by the released bFGF, which was more conducive to angiogenesis and epithelization. Consequently, the CBD–bFGF/BDDM group and the ASCs/CBD–bFGF/BDDM group showed better wound healing qualities than the BDDM group; (3) additional ASCs in ASCs/CBD–bFGF/BDDM could release a broad spectrum of biomolecules to optimize the local immune environment at the wound site (Shen et al., 2016; Sukho et al., 2018), thus attracting endogenous stem/progenitor cell infiltration and promoting ECM synthesis, resulting in an improvement in wound healing. Moreover, a large number of ASCs were loaded on the FBDDM and then transplanted into the wound lesions. The ASCs may act as a cell source that is directly differentiated into endothelial cells or fibrocytes, thus accelerating the formation of granulation tissue at the skin wound. Further in-depth studies are needed to prove these explanations.

Taken together, these results confirm that ASCs/CBD–bFGF/BDDM is a suitable covering graft for diabetic wounds. Nevertheless, there remain several limitations in our study. First, although NDT has suitable tensile strength, regretfully, a tensile strength test of the BDDM was not conducted in our study. According to published literature (Milan et al., 2016; Terzini et al., 2016), NDT could be decellularized without any deterioration of its tensile properties. Considering that our protocol of sectioning NDT into a book-like appearance does not influence the Langer lines of skin (Ottenio et al., 2015), BDDM may have similar tensile properties to NDT. Second, the *in vivo* functions of the implanted ASCs were not elucidated in this study. According to the literature (Frese et al., 2016; Luck et al., 2020), the regenerative effects of these implanted ASCs may be attributed to two aspects. (1) ASCs have multipotent differentiation potential (Frese et al., 2016; Luck et al., 2020), and our *in vitro* results determined that ASCs can differentiate into endothelial cells under the influence of CBD–bFGF/BDDM. We speculate that some implanted ASCs participate in skin wound healing by directly differentiating into endothelial cells, fibroblasts, and so on; and (2) the implanted ASCs secret a series of bioactive substances (cytokines, soluble proteins, growth factors, nucleic acids and membrane-bound vesicles) (Kapur and Katz, 2013; Pallua et al., 2014), which regulate some implanted cells and migrated endogenous progenitor cells involved in the formation of granulation tissue. In the future, further research is needed to reveal the detailed roles of the implanted ASCs. Third, we only comparatively evaluated the *in vivo* efficacies of the DDM, BDDM, CBD–bFGF/BDDM, and ASCs/CBD–bFGF/BDDM on wound healing. Rigorously speaking, we should include a group of diabetic rats without graft covering of their wounds. Because DDM has been authorized for clinical applications, its *in vivo* efficacy for wound healing has been proven at the stage of preclinical research. Thus, we did not include a group of diabetic rats without any skin wound treatment as a negative control in this study. This design is more compliant with the ethical requirements for laboratory animals. Despite these limitations, our results provide a basis for the clinical application of ASCs/CBD–bFGF/BDDM to accelerate diabetic wound healing.

## Conclusion

In summary, a book-shaped decellularized dermal scaffold (termed BDDM) with a high resemblance to NDT in histology, microstructure, and ingredients was developed, and then recombinant bFGF (termed CBD–bFGF) capable of binding collagen was tethered onto this BDDM to improve its angiogenic functions, This functional scaffold (termed CBD–bFGF/BDDM) could largely load stem cells and sustainably release bFGF to stimulate stem cells through endothelial differentiation, Additionally, cells were interleaved into the page gaps of CBD–bFGF/BDDM to develop a novel tissue-engineered graft (termed ASCs/CBD–bFGF/BDDM), which was beneficial for enhancing diabetic wound healing *via* angiogenesis coordination. Furthermore, this study indicates that ASCs/CBD–bFGF/BDDM is a very promising graft for accelerating DFU healing in the future.

## Data Availability Statement

The original contributions presented in the study are included in the article/Supplementary Material, further inquiries can be directed to the corresponding author/s.

## Ethics Statement

The animal study was reviewed and approved by the Animal Ethics Committee of Chenzhou No. 1 People’s Hospital.

## Author Contributions

JL and CC contributed to the conceptualization and supervision. XS, XZ, BC, WS, YCa, YCh, XYL, YH, CL, XRL, and XL contributed to the methodology and validation. XS, XZ, and LJ contributed to data curation and analysis. XS, LJ, and JL contributed to writing (original draft preparation). HL, LJ, and CC contributed to writing (review and editing). HL and JL contributed to funding acquisition. All the authors have read and agreed to the published version of the manuscript.

## Conflict of Interest

The authors declare that the research was conducted in the absence of any commercial or financial relationships that could be construed as a potential conflict of interest.
