# Supplementary figures and images for: Adipose-Derived Stromal Cell-Sheets Sandwiched, Book-Shaped Acellular Dermal Matrix Capable of Sustained Release of Basic Fibroblast Growth Factor Promote Diabetic Wound Healing
Source: Front Cell Dev Biol. 2021 Mar 25;9:646967. doi: 10.3389/fcell.2021.646967 (PMC8027315; doi:10.3389/fcell.2021.646967)

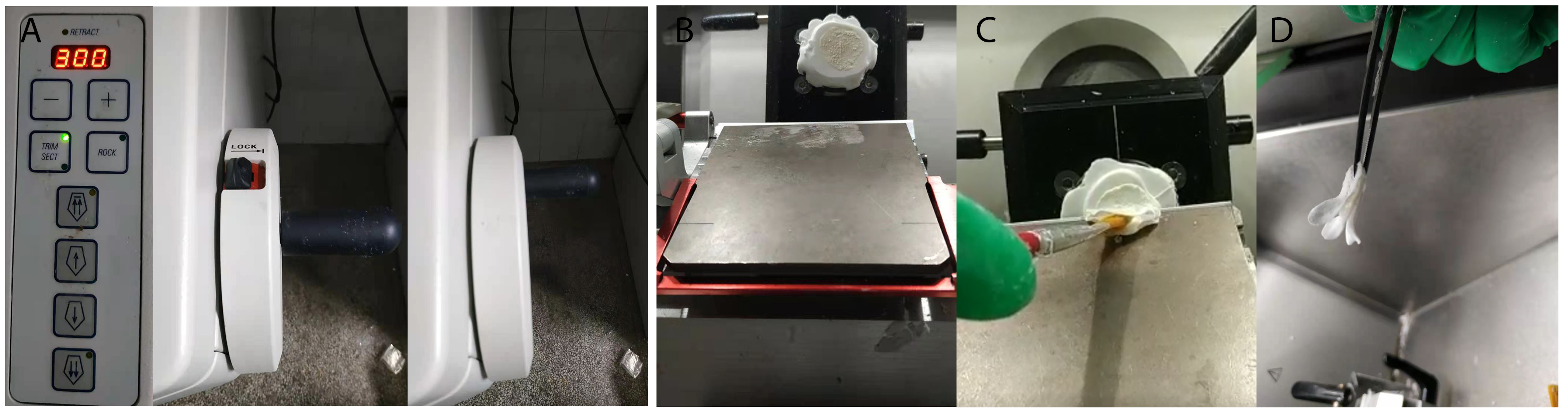

Supplement: Supplementary file 1 [file Image_1.JPEG]
