# Supplementary material for: Adipose-Derived Stromal Cell-Sheets Sandwiched, Book-Shaped Acellular Dermal Matrix Capable of Sustained Release of Basic Fibroblast Growth Factor Promote Diabetic Wound Healing
Source: Front Cell Dev Biol. 2021 Mar 25;9:646967. doi: 10.3389/fcell.2021.646967 (PMC8027315; doi:10.3389/fcell.2021.646967)
Supplement: Supplementary file 2 [file Data_Sheet_1.docx]

**Supplementary Materials**

**Table S1.** Amino acid sequences of CBD-bFGF and NAT-bFGF.

| CBD-bFGF | ﻿MHHHHHHTKKTLRTGGGGSGGGGSGGGGSAAGSITTLPALPEDGGSGAFPPGHFKDPKRLYCKNGGFFLRIHPDGRVDGVREKSDPHIKLQLQAEERGVVSIKGVCANRYLAMKEDGRLLASKCVTDECFFFERLESNNYNTYRSRKYTSWYVALKRTGQYKLGSKTGPGQKAILFLPMSAKS |
| --- | --- |
| NAT-bFGF | AAGSITTLPALPEDGGSGAFPPGHFKDPKRLYCKNGGFFLRIHPDGRVDGVREKSDPHIKLQLQAEERGVVSIKGVCANRYLAMKEDGRLLASKCVTDECFFFERLESNNYNTYRSRKYTSWYVALKRTGQYKLGSKTGPGQKAILFLPMSAKS |

**Table S2.** Primer sequences used for qRT-PCR analysis.

| Gene | Primer sequence | Species |
| --- | --- | --- |
| CD31 | Forward primer 5′- GCTGTCTACTCAGTCATGGCC -3′  Reverse primer 5′- CGTCTCTTTCCTTCTGGATGGTG -3′ | Rat |
| vWF | Forward primer 5′- AGACAGCATCAGGGTCATCAG -3′  Reverse primer 5′- ACTTCACCTTCAGTGGCATCT -3′ | Rat |
| CD144 | Forward primer 5′- ﻿AGAATTTGCCCAGCCCTAC -3′  Reverse primer 5′- ﻿GCGGTATTGTCGTGGTTG -3′ | Rat |
| GAPDH | Forward primer 5′- ﻿AGACAGCCGCATTCCCTTGT -3′  Reverse primer 5′- ﻿TGATGGCAACAATGTCAAGT -3′ | Rat |
| β-actin | Forward primer 5′- ﻿ATCTGGCACCACACCTTC -3′  Reverse primer 5′- ﻿AGCCAGGTCCAGACGCA -3′ | Rat |


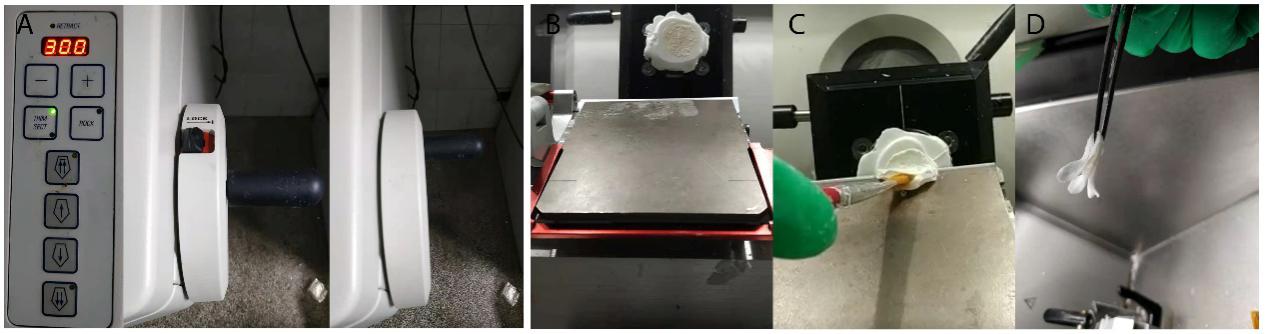


**Figure S1:** The process of book-shaped DDM production : (A) Each page of the BDDM is 300 μm ; (B-D) Each sample was sectioned into 3 pages for BDDM production.
